# Design and Characterization of Aptamers to Antibiotic Kanamycin with Improved Affinity

**DOI:** 10.3390/ijms262211234

**Published:** 2025-11-20

**Authors:** Alexey V. Samokhvalov, Oksana G. Maksimenko, Anatoly V. Zherdev, Boris B. Dzantiev

**Affiliations:** 1 A. N. Bach Institute of Biochemistry, Research Center of Biotechnology, Russian Academy of Sciences, Moscow 119071, Russia; 03alexeysamohvalov09@gmail.com (A.V.S.); zherdev@inbi.ras.ru (A.V.Z.); 2 Institute of Gene Biology, Russian Academy of Sciences, Moscow 119334, Russia; maksog@mail.ru

**Keywords:** aptamers, kanamycin, binding constant, isothermal titration calorimetry, circular dichroism

## Abstract

Aptamers are promising synthetic molecular receptors that bind to specific targets by adopting a unique tertiary structure. However, their selection using standard SELEX protocols often does not allow the achievement of high affinity to the targets. Due to the lack and difficulty of obtaining data on the 3D structure of aptamers and their complexes, the design of known aptamers based on simple rules and software is in demand. The presented work considers the comparative characterization and design of DNA aptamers specific to the antibiotic kanamycin based on complementary interactions and structural motifs (bulges, mismatches, loops) predicted by NUPACK, RNAfold, and UNAFold software. The design included the elimination of non-functional parts of the aptamers and the stabilization of the kanamycin-binding loop. Seven novel aptamers, chosen based on these predictions, were synthesized, and their affinities were measured using an isothermal titration calorimetry technique. The prediction of end stem and hairpin loop structures was confirmed by comparison with circular dichroism data. As a result of sequential design with truncation of unnecessary nucleotides, a novel optimal 42-base-long aptamer was designed and demonstrated a dissociation constant of 109 ± 15 nM, which is 4.7-fold lower than the initial preparation (470 ± 40 nM) and overcomes all known aptamers to kanamycin.

## 1. Introduction

Aptamers are synthetic molecular receptors of a nucleic acid nature. Their advantages as receptors are based on the well-established and easily available methods of nucleic acid synthesis and modification (low-cost synthesis, minimal batch-to-batch variation, strictly localized modifications). Additionally, aptamers are characterized by simple and efficient renaturation, a short single-strand sequence (typically from 25 to 90 nucleotides in length), and low immunogenicity [1,2].

They are mostly obtained through the SELEX method (Systematic Evolution of Ligands by Exponential Enrichment). However, aptamers pre-selected by SELEX are not guaranteed to have an optimal sequence for high-affinity binding with their targets [3,4]. Published descriptions often do not contain details of direct affinity testing, characterization of the influence of non-randomized regions (primer-binding region or capture probes), and the effect of media conditions on the binding properties of selected candidates. Taking this into account, the remodeling of aptamers by post-SELEX modifications of their sequences is widely applied. Such work requires knowledge about the basic structures of initial aptamers and the location of their conserved stabilizing regions and analyte-binding parts [4,5].

Note that in most cases, direct structural data for the aptamers of interest are not available due to the complexity and labor intensity of corresponding techniques such as nuclear magnetic resonance, X-ray crystallography, etc. The possible solution to this is to use computational tools to make predictions of 2D structures from sequences of oligonucleotides, such as NUPACK, UNAFold, and RNAfold, and integrate the results of such estimations with measurements of affinities for the proposed modified aptamers [5,6]. NUPACK additionally allows the prediction of dimerization of proposed aptamers, excluding such variants without experimental testing. It should be noted that these simple computational tools create possible 2D structures primarily based on Watson–Crick pairing, with very limited accounts of non-canonical interactions. Thus, additional experimental characterization is needed to prove the absence of non-canonical structures such as G-quadruplexes, etc. The efficiency of such computational approaches for the remodeling of aptamers was demonstrated for different ligands (dopamine [7], testosterone [8], ochratoxin A [9], aflatoxin B1 [10], etc.). Thus, in this work, we applied structural predictions combined with measurements of the affinities of the proposed modifications of a known DNA aptamer against an analytically relevant target to obtain a variant with improved affinity.

The wide use of antibiotics to treat infections in both humans and animals highlights the importance of their control and the necessity of an evaluation of both the effectiveness of therapy and the safety of consumer products. One such common antibiotic is kanamycin A (KANA), belonging to the aminoglycoside class [11]. KANA has a narrow therapeutic window for safe use and is considered a hard-to-degrade antibiotic that can quickly accumulate in animal by-products (e.g., milk, meat, and eggs). The accumulation of KANA poses a threat to human health due to its ototoxicity and nephrotoxicity, and can lead to the formation of antibiotic-resistant bacterial strains [12,13]. To date, kanamycin is still being used to treat diseases in humans and domestic animals. The World Health Organization recommends its use as an ophthalmological anti-infective agent [14], and the presence of kanamycin in animal-derived products is closely monitored [15]. However, it is gradually being replaced by other aminoglycosides. The low cost and absence of irreversible denaturation make aptamers a promising tool to control pollutants, like KANA, in conditions other than physiological ones.

To date, there have been four published SELEX experiments to produce several kanamycin A-binding DNA aptamers [16,17,18,19]. Song et al. selected aptamers with a low dissociation constant (K_D_) (for example, s11_KANA2 K_D_—78.8 nM) using classic SELEX on an affinity column [16]. K_D_s were measured through the separation of FAM-labeled aptamers on KANA-modified beads. Stoltenburg et al. [17] implemented the capture-SELEX method and evaluated K_D_s through the separation of FAM-labeled aptamers. The K_D_ of the best candidate (s12_13_82) was found to be 3.9 μM. Heemstra et al. utilized a restriction enzyme SELEX (RESELEX) method to isolate KANA-binding aptamers [18]. The K_D_ of the best obtained aptamer (s21_K16) was determined by microscale thermophoresis (MST) to be 340 μM. Zhao et al. conducted an improved version of capture-SELEX to obtain aptamers with stem-loop structures and used isothermal titration calorimetry (ITC) for K_D_ evaluation [19]. Two aptamers with constants s25_KANA6-1 and s25_KANA6-5, with values of 320 and 720 nM, respectively, were identified.

The sequences of the best candidate aptamers chosen for comparison in our study are provided in Table 1. Plaxo et al. published a KANA-binding DNA aptamer [20] that was converted from an RNA aptamer [21], but we excluded it from our study due to its sub-millimolar affinity. The maximum residue level of KANA is equal to or above 0.1 μg/g in various food products of animal origin [15], but considering the complexity of sample preparation, KANA-binding aptamers with better affinity are desirable.

This study focuses on comparing existing aptamers for kanamycin A and selecting a candidate for sequence modification to enhance its binding affinity and remove redundant bases that do not contribute to KANA binding. The NUPACK (https://www.nupack.org/ accessed on 17 October 2025) [22], UNAFold (https://www.unafold.org/ accessed on 17 October 2025), and RNAfold (http://rna.tbi.univie.ac.at/cgi-bin/RNAWebSuite/RNAfold.cgi accessed on 17 October 2025) online computational tools were utilized to predict the 2D structures of the aptamers. Circular dichroism (CD) was then conducted to confirm a stem-like structure. Isothermal titration calorimetry (ITC) allowed direct label-free measurements of complexation in the solution of ligand and receptor to be obtained. Next, the K_D_s of the aptamers were measured using ITC, and the best binding aptamer was chosen for modification. We analyzed the possible structural elements of the aptamer and synthesized seven variants with alterations to these structural motifs. The comparison was conducted to gather information on the role of specific regions of the aptamer on its affinity with KANA, with the goal of identifying the variant with improved binding properties. The combination of computational and experimental approaches enabled the isolation of an aptamer that is 4.7 times more effective than the initial preparation and surpasses all known KANA-binding aptamers in terms of binding constant.

## 2. Results and Discussion

### 2.1. The Comparison of Affinity for Kanamycin-Binding Aptamers

First, to compare the five aptamers selected in different SELEX experiments, we determined their binding parameters using ITC (see Table 1). The goal was to directly measure the complexation between free KANA and the aptamers in solution. Aptamer affinity is sensitive to the composition of the reaction media, including the presence of mono- and divalent metal cations [23]. The selection buffer (SB) from the original selection articles SB1 (for s11_Kana2), SB2 (for s21_K16, s12_13_82), and SB3 (for s25_KANA6-1 and 6-5) were used in ITC experiments to estimate the dissociation K_D_s of each aptamer. Therefore, the implemented analysis of interactions using different SBs pursued three goals: (i) confirming binding under the conditions in which it had already been described, (ii) comparing the measured constants with literature data, (iii) selecting the most promising reagent for further optimizations. The exact composition of the buffers can be found in the Materials and Methods section.

**Figure 1:**
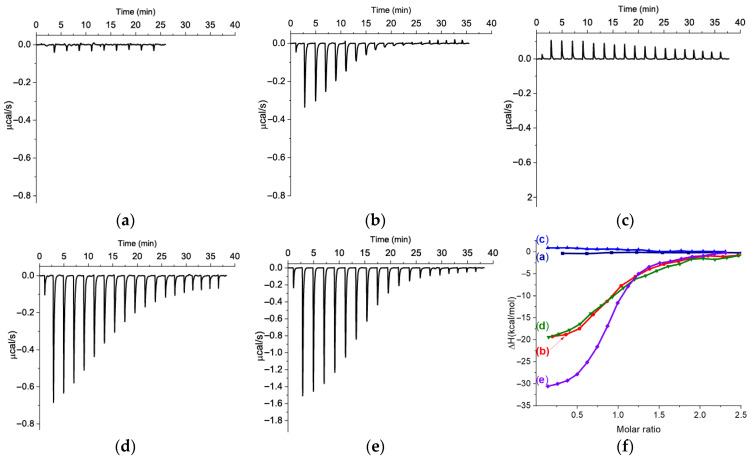
ITC measurements for sequential additions of KANA to the aptamers: (**a**) aptamer s11_Kana2 (20.8 μM)–KANA (300 μM) in SB1; (**b**) aptamer s12_13_82 (8 μM)–KANA (130 μM) in SB2; (**c**) aptamer s21_K16 (30 μM)–KANA (350 μM) in SB2; (**d**) aptamer s25_Kana6-1 (16 μM)–KANA (200 μM) in SB3; (**e**) aptamer s25_Kana6-5 (21.4 μM)–KANA 250 μM) in SB3; and (**f**) the integrated heat plots for KANA interactions with the aptamers (curve (a)—s11_Kana2, curve (b)—s12_13_82, curve (c)—s21_K16, curve (d)—s25_Kana6-1 and curve (e)—s25_Kana6-5).

The sequential addition of KANA to the aptamers solution provides us with the raw data of the resulting heat and plots of enthalpy against molar ratio, showing the integrated heat results for the aptamers–KANA interactions (Figure 1a–e). The thermodynamic parameters are summarized in Table 2. Figure 1f presents a comparison of the obtained integrated plots. It can be observed that the addition of KANA to s11_Kana2 and s21_K16 aptamers did not cause a reliable heat change, suggesting the absence of complexation or very weak binding outside the practical range. Due to the absence of binding events, the use of the 19 injection was considered excessive, and the 9 injections were made during ITC measurements of s11_Kana2 binding with KANA. The window for determining K_D_s by ITC is determined by the receptor (20.8 μM or 30 μM for s11_Kana2 and s21_K16, respectively) concentration in the working cell and is reflected by the parameter “c” (1 < c < 10,000) [24]. The K_D_s weaker than values corresponding to the “c” parameter outside the range by tenfold (c = 0.1) wouldn’t be recognized, and these values are 200 and 300 μM for s11_Kana2 and s21_K16, respectively. In the case of s21_K16, the K_D_ outside the practical range was expected in accordance with the original source (340 μM) [18]. The K_D_ found for s11_Kana2 is in accordance with the data published by Zhao et al. [19], but significantly worse compared with the initial description of this aptamer [16]. Based on the fact that the original K_D_ was obtained with KANA immobilized on magnetic beads, this leads to an agreement with the hypothesis of Zhao et al. [19] that the original K_D_ could be the result of DNA non-specific absorption rather than binding with KANA.

The aptamers s12_13_82, s25_KANA6-1, and s25_KANA6-5 all exhibit exothermic interactions (∆H < 0) with KANA in a 1:1 stoichiometry (N), as shown in Table 2 and Figure 1. Among these aptamers, s25_KANA6-5 shows the greatest change in ∆H, consistent with the data presented by Zhao et al. [19]. However, the obtained absolute ∆H values are greater than those reported, which could be due to a difference in concentration calculations (N in the original study was 0.84 and 0.72, respectively). The K_D_ value for Kana6-5 is in good agreement with the original value, but surprisingly, for Kana6-1, we observed a sevenfold worse K_D_ than previously reported [19].

In the case of aptamer s12_13_82, we observed a K_D_ that was ~10-times better (680 vs. 5100 nM) than in [15]. The initial K_D_ was obtained through ligand-induced strand displacement with further separation of FAM-labeled aptamer fractions, rather than ITC direct measurements of complexation via heat changes. Next, we compared the binding in the SB2 buffer with an alternative low-salt buffer; previously, Zhao et al. [18], in the case of the s25_Kana6-1, showed the presence of salt-dependent binding of KANA under neutral pH, with better affinity in a buffer of low salt content. Herein, we observed similar results in the case of the s12_13_82 aptamer, Figure S1. The binding buffer, containing 50 mM Na^+^ and 3 mM Mg^2+^ (BB), provided 1.4-fold lower K_D_, equal to 475 ± 44 nM. Thus, the BB was used in further experiments to study sequence-dependent binding of the s12_13_82 aptamer with KANA.

### 2.2. Characterization of Structure for Kanamycin-Binding Aptamers Using Circular Dichroism Spectroscopy

**Figure 2:**
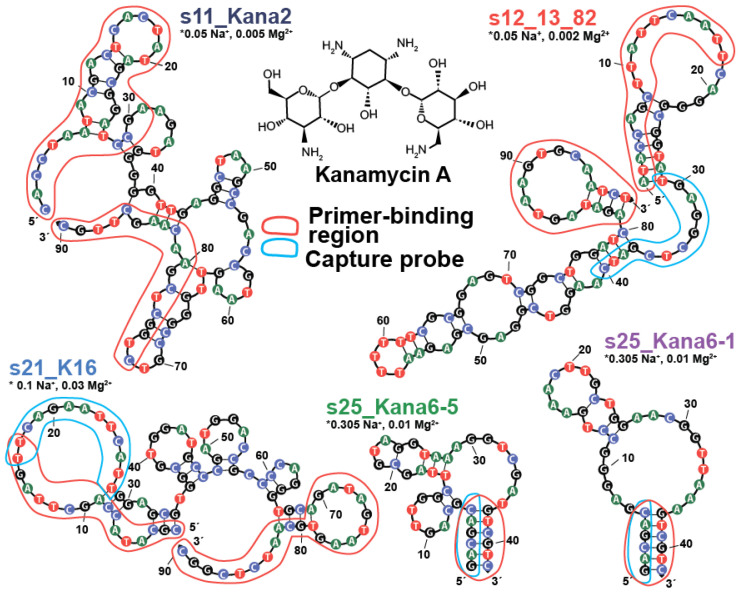
Two-dimensional structures (NUPACK 4.2) of kanamycin-binding aptamers at 25 °C and at specific cationic conditions corresponding to the buffer used during the K_D_ evaluation for this study. The different nucleobases depicted using different colors. The primer-binding regions are marked in red and the capture probe (used during capture-SELEX or RESELEX to immobilize the sequence on a carrier or to enzymatically digest duplexed ones) is marked in blue. The asterisks indicate location of the cationic conditions descriptions.

According to NUPACK predictions, shown in Figure 2, the randomized region of the considered aptamers, which should contain the ligand-binding site, differs in organization. s25_Kana6-1 and s25_Kana6-5 have a defined stem-loop, while s12_13_82 forms an imperfect and highly polymorphic hairpin containing several loops and mismatches. The last two aptamers (s11_Kana2 and s21_K16) do not have a defined, organized structure. No experimental information about their structure is present, except in the case of s25_Kana6-1, where the absence of a G-quadruplex was confirmed by CD spectroscopy [19].

The CD method was chosen for the direct registration of the structural organization of the aptamers. If a ligand induces a change in the base stacking in the transition from one conformational form to another, it causes a change in the CD spectrum [25] and allows the formation of aptamer–ligand complexes to be registered. First, we compared the CD spectra of all aptamers that showed KANA binding in ITC experiments, and, additionally, the s21_K16, to represent the aptamer that failed to bind KANA under the studied conditions (see Figure 1).

**Figure 3:**
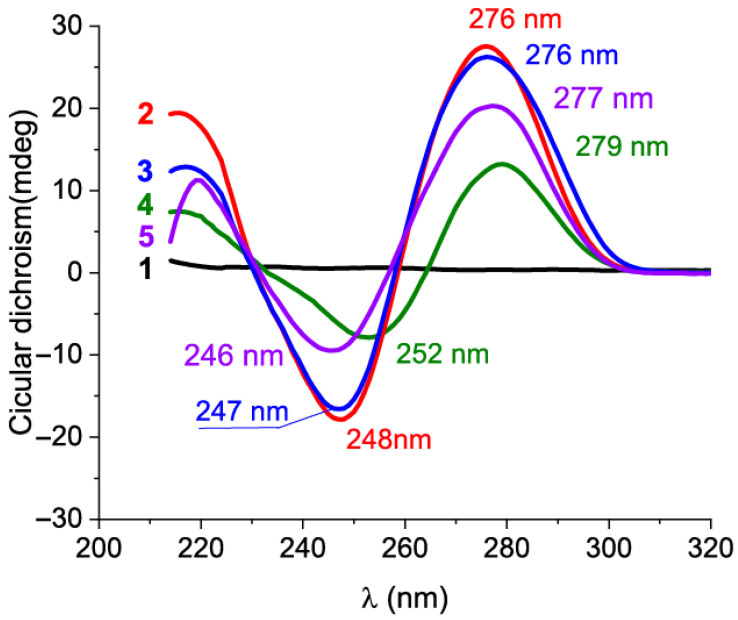
The comparison of CD spectra of (1) 20 μM kanamycin and 2 μM of the aptamers: (2) s12_13_82 in BB, (3) s21_K16 in SB2, (4) s25KANA6-1 in SB3, and (5) s25KANA6-5 in SB3.

A CD spectrum with a positive band around 280 nm and a negative one around 245 nm is considered indicative of the heterogeneous B DNA form [26]. Figure 3 shows the spectra of all aptamers containing negative extrema at ~250 nm and positive extrema at ~276 nm, confirming the possibility of the stem-loop organization of the aptamers. Note that the simulation using NUPACK software (v. 4.0 and 4.2, https://www.nupack.org/ accessed on 17 October 2025) is limited to Watson–Crick and “wobble” G-T interactions and does not predict structures like G-quadruplexes [22]. The resulting CD spectrum integrates contributions of individual nucleotides and 2D conformations, providing a rough estimation that allows for the exclusion of the formation of G-quadruplexes and i-motifs [26].

**Figure 4:**
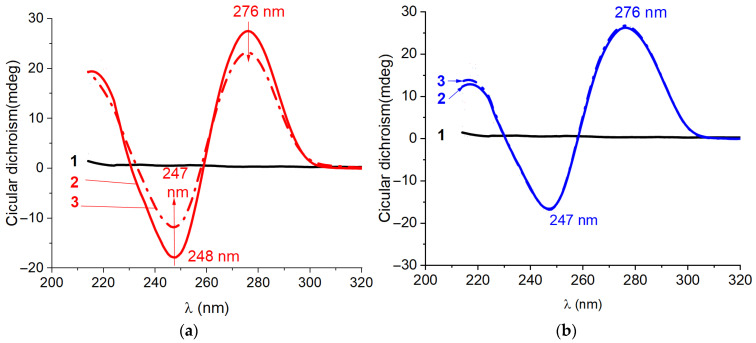

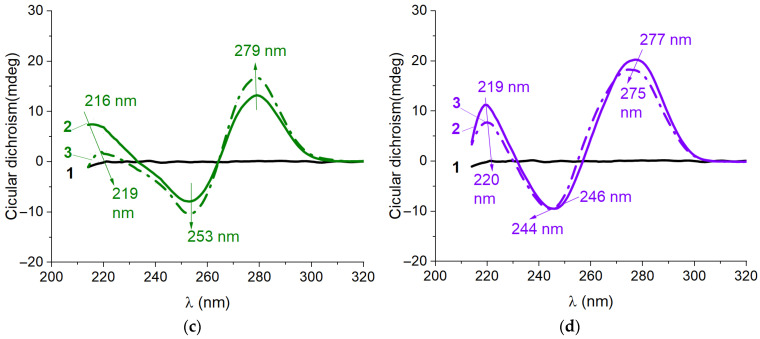
The CD spectra of (1) 20 μM of KANA (black line), (2) 2 μM of aptamer (solid line), and (3) their complex 10 to 1 (dash dot line) for aptamers (**a**) s12_13_82 in BB, (**b**) s21_K16 in SB2, (**c**) s25KANA6-1 in SB3, and (**d**) s25KANA6-5 in SB3.

We measured the spectra of aptamers under a 10-fold excess of KANA. As follows from the obtained K_D_ values (see Section 2.1), this excess of KANA is sufficient to achieve an 80–90% transition of the aptamers (s12_13_82, s25_KANA6-1, and s25_KANA6-5) into the complex. The CD data shown in Figure 4 further confirm the formation of KANA–aptamer complexes.

The spectrum of aptamer s12_13_82 shows a decrease in the amplitudes of both extrema with almost no change in extrema positions after a 10-fold excess of KANA addition, as shown in Figure 4a. Aptamer s21_K16’s spectrum shows no significant change in its CD, as seen in Figure 4b, which was expected based on ITC data. In the case of s25KANA6-1, we observe increases in the amplitudes of all extrema, accompanied by a bathochromic shift in the positive band from 216 to 219 nm. Aptamer s25KANA6-5 shows a decrease in the amplitudes of both positive extrema, accompanied by a hypochromic shift in positive bands at 277 and 246 nm and a bathochromic shift in the positive band at 219 nm.

KANA does not have CD peaks in the studied region. Therefore, it can be unequivocally stated that the change in the aptamer spectrum is associated with the formation of the complex. The aptamers that demonstrate binding in ITC experiments also show distinct changes in CD spectrum in the presence of KANA and retain their stem-loop structure. However, the KANA-induced CD spectrum change was unique for each aptamer.

Based on ITC and CD data, aptamers s25_Kana6-5 and s12_13_82 are perceived as prospective candidates for further study and have not yet been characterized as receptors for analytical purposes. The 2D structure of aptamer s12_13_82 contains obvious extra elements, such as primer-binding regions and dangles in the capture probe, as shown in Figure 2. Additionally, multiple mismatches and bulges were predicted in the structure that could destabilize the expected KANA-binding pocket. These findings indicate that improvement of s12_13_82 binding affinity can be achieved by the truncation and stabilization of the predicted elements. Therefore, we have further considered modifications of this aptamer.

### 2.3. Modification of Kanamycin Aptamer Based on 2D Model and ITC Measurements

#### 2.3.1. The Truncation of Initial Aptamers and Modification of End-Stem Region

**Figure 5:**
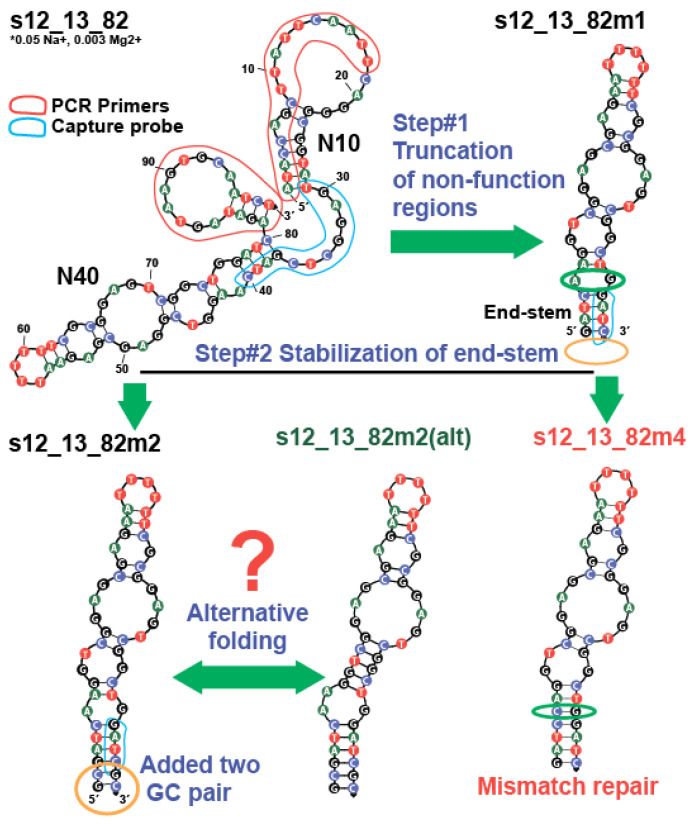
The 2D structures (NUPACK 4.2) of aptamer s12_13_82 at specific cationic condition and its modification at 25 °C. The different nucleobases depicted using different colors. The added GC pairs are marked by orange circles, and corrected A-G mismatch is marked by green circle. The primer-binding regions are marked in red and the capture probe is marked in blue. The asterisk indicates location of the cationic condition description.

After characterizing the K_D_ of s12_13_82 aptamer and confirming its stem-loop structure, we conducted post-SELEX optimization of its sequence based on NUPACK (v. 4.2) prediction and the initial design by Stoltenburg et al. [17]. The aptamer sequence consisted of two primer-binding regions (marked red), an 11 nt-long “capture probe” (marked blue), and two randomized regions (N10 and N40) (see Figure 5). The forward primer-binding region forms a hairpin with the N10 region, and the backward primer-binding region forms a hairpin with itself. The capture probe is the site of the formation of an intermolecular duplex with a complementary strand. In order for a sequence to be selected during initial capture-SELEX, the presence of KANA in the media has to disrupt the intermolecular duplex by forming a more energetically favorable conformation, such as an intramolecular duplex [27]. An intramolecular duplex can be observed in the 37–40 nt region (marked blue) in Figure 5.

**Figure 6:**
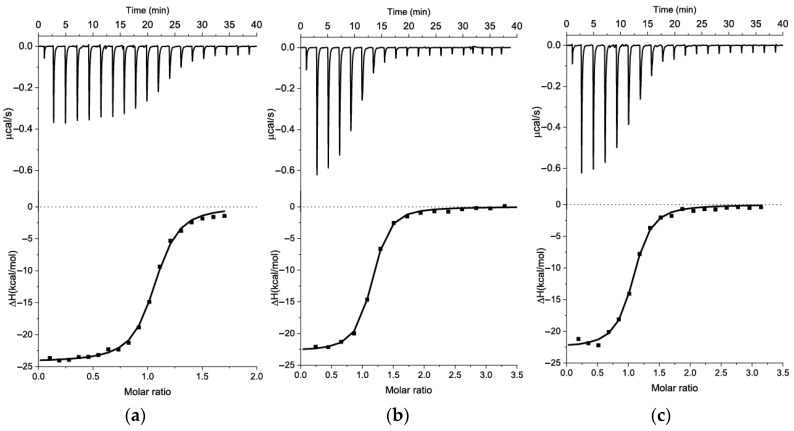
ITC measurements for sequential additions of KANA to the aptamers in BB and the integrated heat plots for the following interactions: (**a**) s12_13_82m1 (8.7 μM)–KANA (75 μM); (**b**) s12_13_82m2 7.4 μM)–KANA (150 μM); and (**c**) s12_13_82m4 (9.4 μM)–KANA (150 μM).

In the first step, we truncate the primers and associated hairpins, as well as the dangling end of the capture probe. The dangle seems to destabilize the stem at the 37–40 nt position, potentially affecting the stability of the overall structure between 37 and 80 nucleotides, containing the N40 region with an expected KANA binding site. The new 44-nt long variant is named s12_13_82m1, and its K_D_ was characterized using ITC (see Figure 6a).

The s12_13_82m1 aptamer (Figure 6a) shows a 4.7-fold increase in K_D_ compared to the original aptamer, as shown in Table 3. The s12_13_82m1 sequence, containing nucleotides from the 37th to the 80th, maintained the enhanced KANA affinity. However, it is structurally diverse and includes an interior stem-loop, multiple mismatches, and a hairpin, as shown in Figure 5. Our first step was to stabilize the end-stem region, which holds the entire structure together, by adding two GC pairs to its end (highlighted in orange in Figure 5) or correcting an A-G mismatch (highlighted in green in Figure 5), as seen in variants m2 and m4 in Figure 5. We carried out ITC measurements for both m2 (Figure 6b) and m4 (Figure 6c) variants, with the results summarized in Table 3.

Interestingly, the addition of two GC pairs did not influence the K_D_, suggesting that the end-stem is already sufficiently stable under the tested conditions for optimal binding. Moreover, the replacement of A in the 41st position with C, which was performed to correct the mismatch with G76, led to a two-fold decrease in K_D_. This suggests that the mismatch between the 41A and G76 nucleotides played a role in efficient KANA binding. It is possible that the flexibility of this region is necessary, or that a different folding pattern than the one predicted for s12_13_82m2 (Figure 5) is correct. This folding discrepancy was studied by comparing 2D structures using NUPACK v4.2 and v4.0, UNAFold, and RNAfold, as shown in Figure S2. UNAFold and NUPACK v4.0 predict the same structure (Figure S2b,c and Figure 5 (variant s12_13_82m2(alt)), while NUPACK v4.2 (Figure 5, variant s12_13_82m2) and RNAfold (Figure S2d) provide some differing folding variants. Base-pairing probabilities below 50%, as given by NUPACK and RNAfold, indicate high structural uncertainties in the regions from 42 to 47 and from 70 to 75 nucleotides, that are involved in duplex formation The structure of these regions is presented in Figure 5 (variant s12_13_82m2(alt)) and Figure S2b,c using a UNAFold and NUPACK v4.0 2D model, which shows a long duplex region with wobble GT pairs. However, the true structural organization of this region remains uncertain.

#### 2.3.2. The Modification of the Aptamer Hairpin and Interior Loop Organization

**Figure 7:**
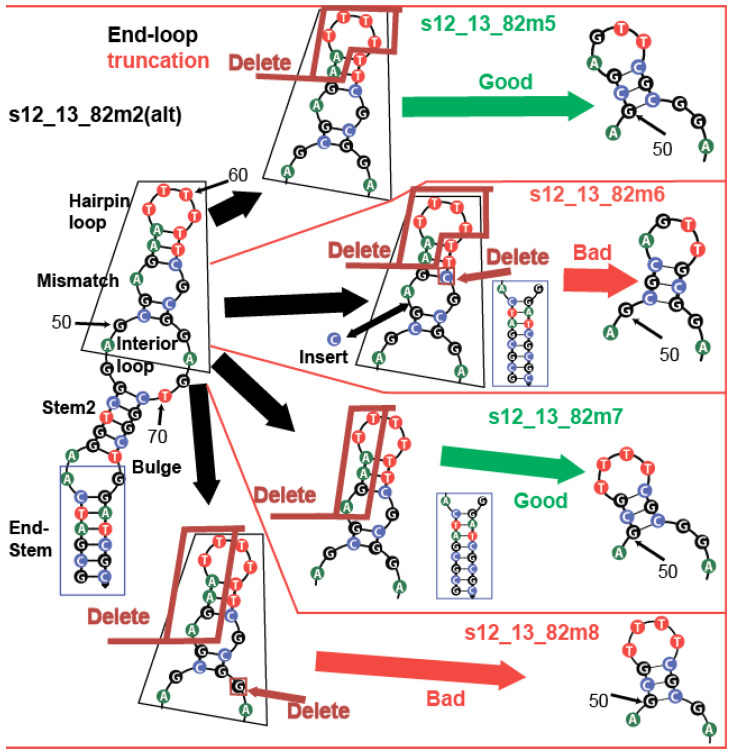
Two-dimensional structures (NUPACK 4.0) of kanamycin-binding aptamer s12_13_82 and its modification at 25 °C. The different nucleobases depicted using different colors. The primer-binding regions are marked red and the capture probe, used during capture-based SELEX to immobilize the sequence on a carrier, is marked blue.

The common view is that whenever aptamers are larger than their target (like in the case of kanamycin A), they tend to incorporate it into their structure [3]. Interaction with a small molecule should not require many nucleobases, with most of them being necessary for positioning the few interacting residues correctly. This idea is supported by known NMR-based binding models of aptamer–ligand complexes, such as aflatoxin B1 [10] and ochratoxin A [28]. If only a few nucleobases directly interact with KANA, is such a complex 2D structure (shown in Figure 7, on the left) necessary?

**Figure 8:**
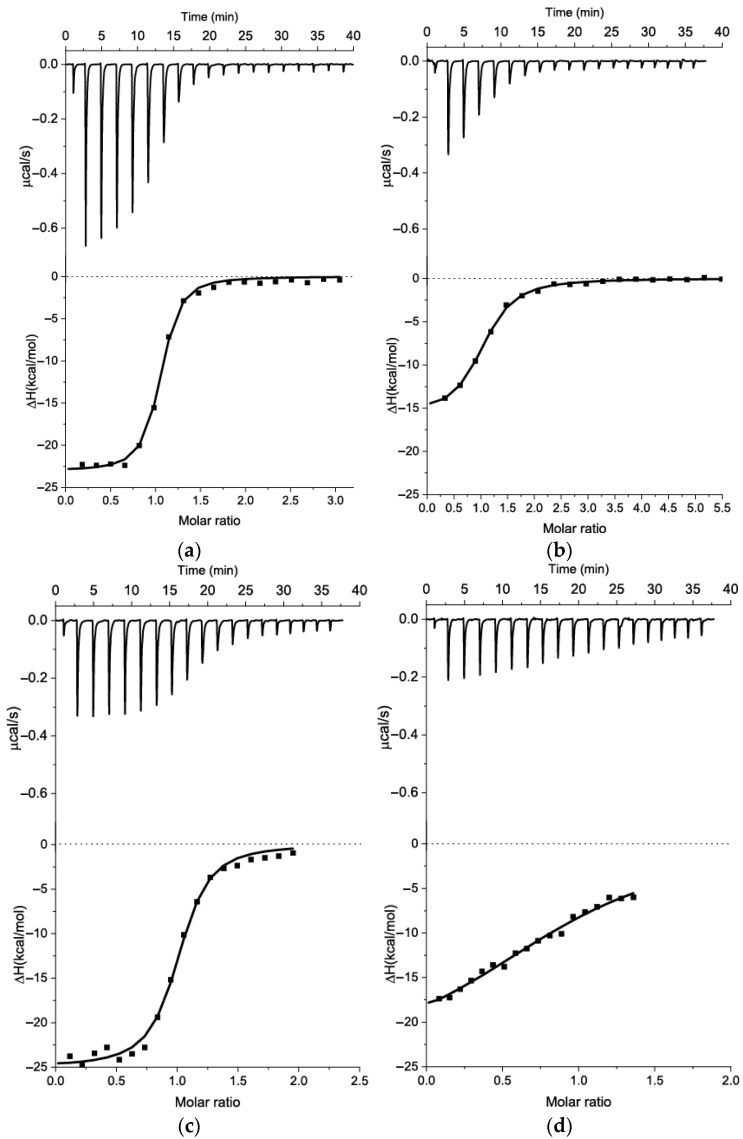
ITC measurements for sequential additions of KANA to the aptamers in BB and the integrated heat plots for the following interactions: (**a**) s12_13_82m5 (9.7 μM)–KANA (150 μM); (**b**) s12_13_82m6 (5.4 μM)–KANA (150 μM); (**c**) s12_13_82m7 (7.6 μM)–KANA (75 μM); and (**d**) s12_13_82m8 (9.4 μM)–KANA (150 μM).

To address this question, we truncated the predicted hairpin loop region between the 51st and 66th nucleotides to a smaller hairpin in two ways—one with a change (13_82m5) and the other with preservation (13_82m6) of the interior loop composition, as shown in Figure 7 (on the right). We conducted K_D_ estimation using ITC for both variants (Figure 8a,b) and obtained binding parameters, as shown in Table 4.

Both variants retain KANA-binding abilities after the truncation of the predicted hairpin region, as seen in Table 4. The variant s12_13_82m6, which retains the same inter-loop as the initial s12_13_82m2 variant (Figure 7), exhibits a 5-fold decrease in KD and a 1.4-fold decrease in entropy, despite the additional 2GC pair stabilizing its end duplex. On the other hand, the 13_82m5 variant with an interloop organization different from that predicted for the initial aptamer in Figure 2 and Figure 5 shows the same binding characteristics as the initial s12_13_82m2 variant. Based on NUPACK and RNAfold predictions (see Figure S2b,d), the G50 can be located either in the interior loop or in the stem that stabilizes the hairpin. The obtained K_D_ values (Table 4) allow a hypothesis to be generated that the organization of the junction between the hairpin and interior loop observed in s12_13_82m5 and m7 variants (Figure 7) is correct; the same organization was predicted by RNAfold for the initial aptamer (Figure S2d).

To confirm that (i) there is no alternative assembly of the hairpin and (ii) the unpaired base of the hairpin loop does not interact directly with KANA, we changed AG in the loop to TT. Additionally, we added two GC pairs at the end duplex (see s12_13_82m7 in Figure 7) to demonstrate that this change does not influence the K_D_. The ITC data (Figure 8c) demonstrated that s12_13_82m7 had the same affinity as s12_13_82m5, suggesting that the loop of the hairpin does not directly interact with KANA. We hypothesized that the predicted hairpin provides the correct orientation of nucleobases G50 and C65 for the formation of the KANA-binding pocket in the interior loop region. Deletion of G66 in the interior loop, as seen in ITC data in Figure 8d, led to a 22-fold decrease in K_D_ and a 1.4-fold decrease in interaction enthalpy. We did not completely disrupt the binding pocket, but by making it more cramped, we hindered its optimal assembly. This supports the position of the binding pocket in the interior loop. Using an RNA composer (https://rnacomposer.cs.put.poznan.pl/ accessed on 17 October 2025) [29], we transformed the 2D structure of s12_13_82m7 (Figure 7) into 3D RNA, then changed the uracil bases into thymine bases and visualized the end product using w3DNA (http://web.x3dna.org/ accessed on 17 October 2025) [30]. The obtained hypothetical 3D structure of s12_13_82m7 is presented in Figure S3. 

### 2.4. Modification of Kanamycin Aptamer of Sequences

Various strategies have been developed to overcome the drawbacks of pre-selected aptamers in the application by modifying their sequence but not chemically changing the natural building blocks [4,5]. Other more sophisticated and very promising approaches include the use of computational methods based on machine learning methods [31,32], structure and interaction prediction via docking and molecular dynamics [32,33], or using the solution of the actual 3D structure of aptamer–ligand complexes [9]. Despite that, more straightforward approaches relying on brute force or researchers’ insights coupled with the manual curation clustering [34] of enriched sequence families or the analysis of predicted 2D structures [6,7,8] are actively and successfully utilized to obtain shorter aptamer sequences, maintaining the same or even better affinity and specificity against the target compared with parent sequences [4,5,31].

The cutting of primer-binding regions is a reasonable first step in aptamer modification. Generally, recognition of the target compound is considered to be independent of the primer-binding regions. The major involvement of primers in the potential ligand-binding site can be seen in the NUPACK-predicted structures of the s11_Kana2 [16] and s21_K16 [18] aptamers, shown in Figure 2. Both aptamers had variants without primer-binding regions characterized in the original studies, but the published K_D_ values of truncated variants were comparable to the values for full aptamers. Therefore, we only include their full-length variant in our study.

In the case of the s25_Kana6-1 and s25_Kana6-5 aptamers, a sequence library with a predetermined hairpin structure was used, limiting the primer-binding region’s participation in an aptamer structure to the role of stabilizing the stem. A previous study carried out sequence clustering to determine the conserved regions in both aptamers. Furthermore, they conducted point mutations of this region at two positions and observed a loss of affinity in the case of s25_Kana6-1. However, no change in the Mfold-predicted 2D structure was observed [19].

For the s12_13_82 aptamer, no previous attempts to truncate its sequence, excluding the primer-binding regions, were conducted. The consensus region was unclear, even after the clustering of similar sequences provided after a SELEX experiment [16].

Another question is the selectivity of the considered aptamers. To characterize the selectivity of the aptamer and its modified variants, we conducted an ITC binding study of the initial s12_13_82 and modified s12_13_82m7 aptamers (Figures S4 and S5) with several common aminoglycosides (gentamicin, neomycin, and streptomycin). No cross-reactivity was observed with neomycin and streptomycin. In the case of gentamicin, ITC registered a small (less than −3 kcal/mol versus >−20 kcal/mol with KANA) concentration-dependent heat change, which was not enough to calculate the affinity. These results indicate weaker binding of orders of magnitude of the aptamer with gentamicin than with KANA. No difference in specificity between s12_13_82 and s12_13_82m7 was observed. Nikolaus et al. [35] carried out the ligand-induced elution of the aptamer from a small capture oligonucleotide using different aminoglycosides and reported cross-reactivity with aminoglycosides from the kanamycin subgroup (specifically kanamycin B, apramycin, tobramycin) and netilmicin.

By modifying the KANA-binding aptamer based on its predicted 2D structure, we obtained the aptamer variants (m1, m2, m5, m7) with an approximate K_D_ of 100 nM to KANA. The obtained K_D_ is 4.7-fold better than the original aptamer and better than the K_D_ of other published aptamers. Through modification, we obtained a shorter 42 nucleotide long aptamer variant compared to the original 98 nucleotide long aptamer. The improved affinity and shorter length make this new aptamer variant a promising choice for application as a bioreceptor for kanamycin A detection.

## 3. Materials and Methods

### 3.1. Reagents and Sample Preparation

Kanamycin A sulfate (PHR), neomycin (VETRANAL), gentamicin sulfate (590 I.U. per µg), tris(hydroxymethyl)aminomethane (99%), and sodium acetate (99.5%) were from Sigma-Aldrich (Burlington, MA, USA); magnesium acetate (99%) was from Helicon (Moscow, Russia). Streptomycin sulfate (720 I.U. per µg) was from AppliChem GmbH (Darmstadt, Germany). Potassium chloride (99%) and calcium chloride (97%) were from Loba Chemie (Mumbai, India). 2-(N-morpholino)ethanesulfonic acid (MES) was from Honeywell (Morristown, NJ, USA). All chemicals were analytical grade or chemical reagent grade. A Simplicity Milli-Q^®^ system from Millipore (Darmstadt, Germany) was used to obtain ultrapure water for buffers and solutions of reagents.

All oligonucleotides (see Table 1) were custom-synthesized and purified (>95%) by Syntol (Moscow, Russia). Stock solutions of oligonucleotides were prepared by dissolving their lyophilized powder in deionized water. The concentrations of oligonucleotides were measured using a NanoDrop 2000 microvolume spectrophotometer (Thermo Scientific, Waltham, MA, USA), based on optical density at 260 nm. Stock solutions were stored at −20 °C. Aptamer working solutions were prepared just before measurements by diluting stock solutions in an appropriate buffer. Antibiotics were dissolved in mQ water to a concentration of 40 mg/mL and stored as stocks at −20 °C. Working solutions of antibiotics were freshly prepared in the appropriate buffer for each measurement. We did not observe reliable differences between the prepared aliquots of antibiotic solutions during the series of ITC experiments.

### 3.2. The Measurements of K_D_ for Kanamycin-Binding Aptamers Using ITC

Isothermal titration calorimetry was performed using the MicroCal PEAQ-ITC titration calorimeter (Malvern Panalytical, Morburn, UK) at 25 °C in buffers used for the SELEX experiment, as described below:

SB1—selection buffer 1 (20 mM Tris–Acetate, 50 mM NaAcetate, 5 mM KCl, 5 mM Mg(CH_3_COO)_2_, pH 8.0) [16];

SB2—selection buffer 2 (20 mM Tris-Acetate, 100 mM NaAcetate, 2 mM Mg(CH_3_COO)_2_, 5 mM KCl, 1 mM CaCl_2_, pH 7.6) [17,18];

SB3—selection buffer 3 (50 mM MES-NaOH, 300 mM NaAcetate, 10 mM Mg(CH_3_COO)_2_, 5mM KCl, pH 7.0) [19];

For s12_13_82 and its variants, experiments were carried out in optimized binding buffer (BB)—binding buffer (20 mM Tris–Acetate, 50 mM NaAcetate, 3 mM Mg(CH_3_COO)_2_, pH 7.4).

All buffers were filtered and degassed using a Pyrex^®^ Vacuum filtration system (Merck, Darmstadt, Germany). Before ITC measurements, each aptamer working solution was heated for 7 min at 90 °C and then cooled to room temperature. A NanoDrop2000 microvolume spectrophotometer (Thermo Scientific, Waltham, MA, USA) was used to verify the concentrations of the aptamers.

Initially, 280 μL of the aptamer was added into the reaction cell, and then the titration syringe was loaded with 40 μL of KANA. For aptamer s11_Kana2, the experiment consisted of 9th successive 3.4 μL injections (except for the first 0.4 μL injection). Total injection time was 26 min. In all other cases, the experiments consisted of 19 successive 2.0 μL injections (except for the first 0.4 μL injection) of KANA every 135 s with constant stirring at 750 rpm. Total injection time was approximately 40 min.

To exclude the thermal effects associated with dilutions, control experiments with an appropriate buffer instead of the aptamer solutions were implemented, and the values obtained were used to correct the raw ITC data.

The equilibrium dissociation constant (K_D_), stoichiometry (N), enthalpy (∆H), entropy (∆S), and the Gibbs energy (∆G) of the aptamer–KANA interactions were determined by the MicroCal PEAQ-ITC Analysis Software v1.41 (Malvern Panalytical, Malvern, UK) using the one-site binding model.

### 3.3. Circular Dichroism Spectra Measurements at Equilibrium

All measurements were carried out using a Chirascan spectrometer (Applied Photophysics, Mole Valley, UK) with a 1.0 nm bandwidth and 3 ns integration time. The CD and absorbance spectra of the aptamer, KANA, and their complexes in BB (s12_13_82), SB2 (aptamer s21_K16), and SB3 (S25_KAN6-1, S25_KAN6-5) were obtained at 25 °C in the range from 214 to 340 nm. CD was measured as ellipticity [mdeg].

First, 4 μM stock solutions of aptamers and 40 μM solutions of KANA in the appropriate buffer were prepared. CD spectra under equilibrium conditions were measured using the following mixtures: (1) 400 μL of the KANA or aptamer solution mixed with an equal volume of buffer to achieve a final concentration of 2 μM of aptamer or 20 μM of KANA; (2) 400 μL of the aptamer mixed with an equal volume of KANA in the appropriate buffer at a molar ratio of 1 to 10. The final volume of 800 μL was added to the quartz cuvette with a 5 mm path length.

The CD data were analyzed using the Pro-Data Chirascan package v.4.2.20 (Applied Photophysics, Mole Valley, UK) and processed using Origin 8.1 (Origin Lab, Northampton, MA, USA).

## 4. Conclusions

Understanding the relationships between the structural organization and affinity of aptamers is crucial for their rational design and the full realization of their potential as bioreceptors. In this study, we compared the affinity of known kanamycin A-binding DNA aptamers and undertook the optimization of the aptamer with the highest affinity. Through affinity measurements using ITC, structural analysis via CD, and structure prediction with NUPACK, UNAFold, and RNAfold, we obtained a new variant of kanamycin A-binding aptamer with the highest confirmed affinity among published anti-kanamycin A aptamers. This work serves as a foundation for further applications of the proposed aptamer for different biosensors.

## Figures and Tables

**Table 1:** Sequences of the best kanamycin-binding DNA aptamer candidates selected in published SELEX experiments.

Indications	Aptamers (5′-3′)																																									
s11*_Kana2	1	2	3	4	5	6	7	8	9	10	11	12	13	14	15	16	17	18	19	20	21	22	23	24	25	26	27	28	29	30	31	32	33	34	35	36	37	38	39	40	41	42
	C	A	C	C	T	A	A	T	A	C	G	A	C	T	C	A	C	T	A	T	A	G	C	G	G	A	T	C	C	G	A	A	G	A	T	G	G	G	G	G	T	T
	43	44	45	46	47	48	49	50	51	52	53	54	55	56	57	58	59	60	61	62	63	64	65	66	67	68	69	70	71	72	73	74	75	76	77	78	79	80	81	82	83	84
	G	A	G	G	C	T	A	A	G	C	C	G	A	C	C	G	T	A	A	G	T	T	G	G	G	C	C	G	T	C	T	G	G	C	T	C	G	A	A	C	A	A
	85	86	87	88	89	90																																				
	G	C	T	T	G	C																																				
s12_13_82	1	2	3	4	5	6	7	8	9	10	11	12	13	14	15	16	17	18	19	20	21	22	23	24	25	26	27	28	29	30	31	32	33	34	35	36	37	38	39	40	41	42
	A	T	A	C	C	A	G	C	T	T	A	T	T	C	A	A	T	T	C	A	G	G	G	C	G	G	T	A	T	G	A	G	G	C	T	C	G	A	T	C	A	A
	43	44	45	46	47	48	49	50	51	52	53	54	55	56	57	58	59	60	61	62	63	64	65	66	67	68	69	70	71	72	73	74	75	76	77	78	79	80	81	82	83	84
	G	G	T	C	G	G	A	G	C	G	A	G	A	A	T	T	T	T	T	T	C	G	C	G	G	A	G	T	C	G	G	C	T	G	G	A	T	C	A	G	A	T
	85	86	87	88	89	90	91	92	93	94	95	96	97	98																												
	A	G	T	A	A	G	T	G	C	A	A	T	C	T																												
s21_K16	1	2	3	4	5	6	7	8	9	10	11	12	13	14	15	16	17	18	19	20	21	22	23	24	25	26	27	28	29	30	31	32	33	34	35	36	37	38	39	40	41	42
	C	G	C	A	T	A	C	C	A	G	C	T	T	A	G	T	T	C	A	G	A	A	T	T	C	A	T	T	G	G	A	G	C	G	T	G	G	C	G	T	G	G
	43	44	45	46	47	48	49	50	51	52	53	54	55	56	57	58	59	60	61	62	63	64	65	66	67	68	69	70	71	72	73	74	75	76	77	78	79	80	81	82	83	84
	A	T	G	C	C	C	G	A	T	G	A	A	C	C	G	C	C	C	C	A	G	G	G	T	G	C	A	G	A	T	A	G	T	A	A	G	T	G	C	A	A	T
	85	86	87	88	89	90																																				
	C	T	C	G	G	C																																				
s25_Kana6-1	1	2	3	4	5	6	7	8	9	10	11	12	13	14	15	16	17	18	19	20	21	22	23	24	25	26	27	28	29	30	31	32	33	34	35	36	37	38	39	40	41	42
	G	A	C	G	A	C	G	A	G	G	G	C	C	T	G	A	A	A	C	T	T	G	C	T	G	G	A	A	C	G	G	T	T	A	A	A	G	T	C	G	T	C
s25_Kana6-5	1	2	3	4	5	6	7	8	9	10	11	12	13	14	15	16	17	18	19	20	21	22	23	24	25	26	27	28	29	30	31	32	33	34	35	36	37	38	39	40	41	42
	G	A	C	G	A	C	G	C	A	G	T	T	G	G	C	T	T	A	G	C	G	T	A	G	G	T	A	A	A	G	G	T	C	G	A	T	G	T	C	G	T	C

* The prefix “s11_” is to indicate the year of the aptamer SELEX. Primer-binding region, marked in red. A capture probe used for library Immobilization during SELEX, marked in blue.

**Table 2:** Comparison of the obtained ITC results and the literature data about kanamycin A binding with the studied DNA aptamer candidates.

Isothermal Calorimetry							^2^ K_D_ (μM)
Aptamer	Medium	N	∆H (kcal/mol)	∆S × T (kcal/mol)	∆G (kcal/mol)	K_D_ (μM)	
s11_Kana2	SB1	NA ^1^	−0.4	NA			~0.08 (FAM-labeled aptamer to target-immobilized beads) [16]
s12_13_82	SB2	0.91	−21.7 ± 0.5	−13.3	−8.42	0.68 ± 0.08	5.1 (ligand-induced displacement of FAM-labeled aptamer) [17]
	BB	1.02	−21.9 ± 0.4	−13.3	−8.64	0.47 ± 0.04	
s21_K16	SB2	NA	1.4	NA			340 (MST) [18]
s25_Kana6-1	SB3	0.95	−23.1 ± 0.7	−15.4 ± 0.6	−7.7	2.23 ± 0.24	0.32 ± 0.1 (ITC) [19]
s25_Kana6-5		0.90	−32.4 ± 0.2	−24.2	−8.28	0.87 ± 0.04	0.72 ± 0.02 (ITC) [19]

^1^ NA—Not available. ^2^ reported K_D_.

**Table 3:** Parameters for KANA binding with the modified variants of the DNA aptamer s12_13_82.

Isothermal Calorimetry						
Aptamer	Medium	N	∆H (kcal/mol)	∆S × T (kcal/mol)	∆G (kcal/mol)	K_D_ (nM)
s12_13_82m1	BB	1.03	−24.2 ± 0.2	−14.7	−9.60	96 ± 8.7
12_13_82m2		1.07	−22.2 ± 0.2	−12.6	−9.54	101 ± 8.0
s12_13_82m4		1.03	−22.6 ± 0.4	−13.5	−9.15	199 ± 24.9

**Table 4:** Parameters for kanamycin binding with the modified DNA aptamer s12_13_82.

Isothermal Calorimetry						
Aptamer	Medium	N	∆H (kcal/mol)	∆S × T (kcal/mol)	∆G (kcal/mol)	K_D_ (nM)
s12_13_82m5	BB	0.99	−23.1 ± 0.3	−13.6	−9.52	106 ± 8.7
12_13_82m6		0.99	−15.9 ± 0.4	−7.34	−8.58	519 ± 48.4
s12_13_82m7		0.98	−24.5 ± 0.3	−15.0	−9.50	109 ± 14.9
12_13_82m8		0.90	−18.5 ± 0.7	−10.8	−7.76	2200 ± 352

## Data Availability

The original contributions presented in this study are included in the article/Supplementary Materials. Further inquiries can be directed to the corresponding author.

## Supplementary Materials

The following supporting information can be downloaded at https://www.mdpi.com/article/10.3390/ijms262211234/s1.

## Abbreviations

The following abbreviations are used in this manuscript:

MST	Microscale thermophoresis
ITC	Isothermal titration calorimetry
CD	Circular dichroism
KANA	Kanamycin A
SB	Selection buffer
BB	Binding buffer
