# Supplementary material for: Design and Characterization of Aptamers to Antibiotic Kanamycin with Improved Affinity
Source: Int J Mol Sci. 2025 Nov 20;26(22):11234. doi: 10.3390/ijms262211234 (PMC12653048; doi:10.3390/ijms262211234)
Supplement: Supplementary file 1 [file ijms-26-11234-s001.zip › ijms-3964757-supplementary.pdf]

Supplementary Materials

Design and Characterization of Aptamers to Antibiotic Kanamycin with Improved Affinity

Alexey V. Samokhvalov [1], Oksana G. Maksimenko [2], Anatoly V. Zherdev [1] and Boris B. Dzantiev [1,*]

[1] A. N. Bach Institute of Biochemistry, Research Center of Biotechnology, Russian Academy of Sciences, Moscow 119071, Russia; 03alexeysamohvalov09@gmail.com (A.V.S.); zherdev@inbi.ras.ru (A.V.Z.)
[2] Institute of Gene Biology, Russian Academy of Sciences, Moscow 119334, Russia; maksog@mail.ru
* Correspondence: dzantiev@inbi.ras.ru; Tel.: +7-495-954-31-42

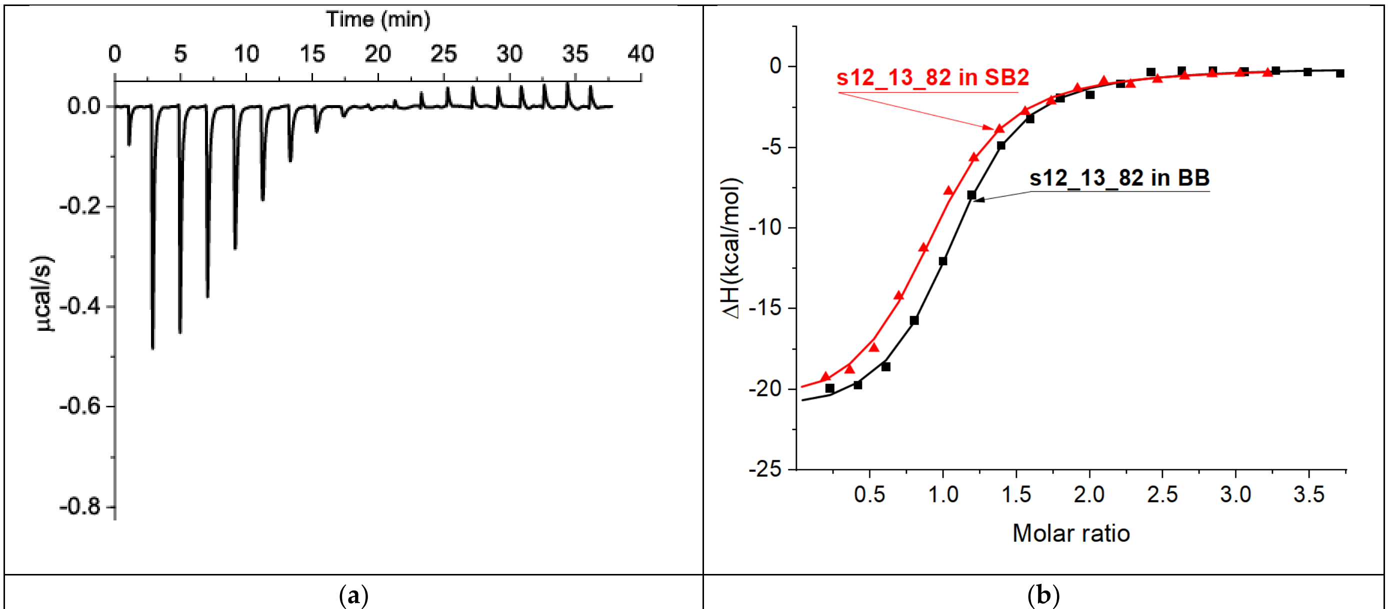

(**a**) (**b**)

Figure S1. ITC measurements for (**a**) sequential additions of KANA (150 µM) to the aptamer s12_13_82 (8 µM) and (**b**) the comparison of integrated heat plots for interaction in SB2 and BB.

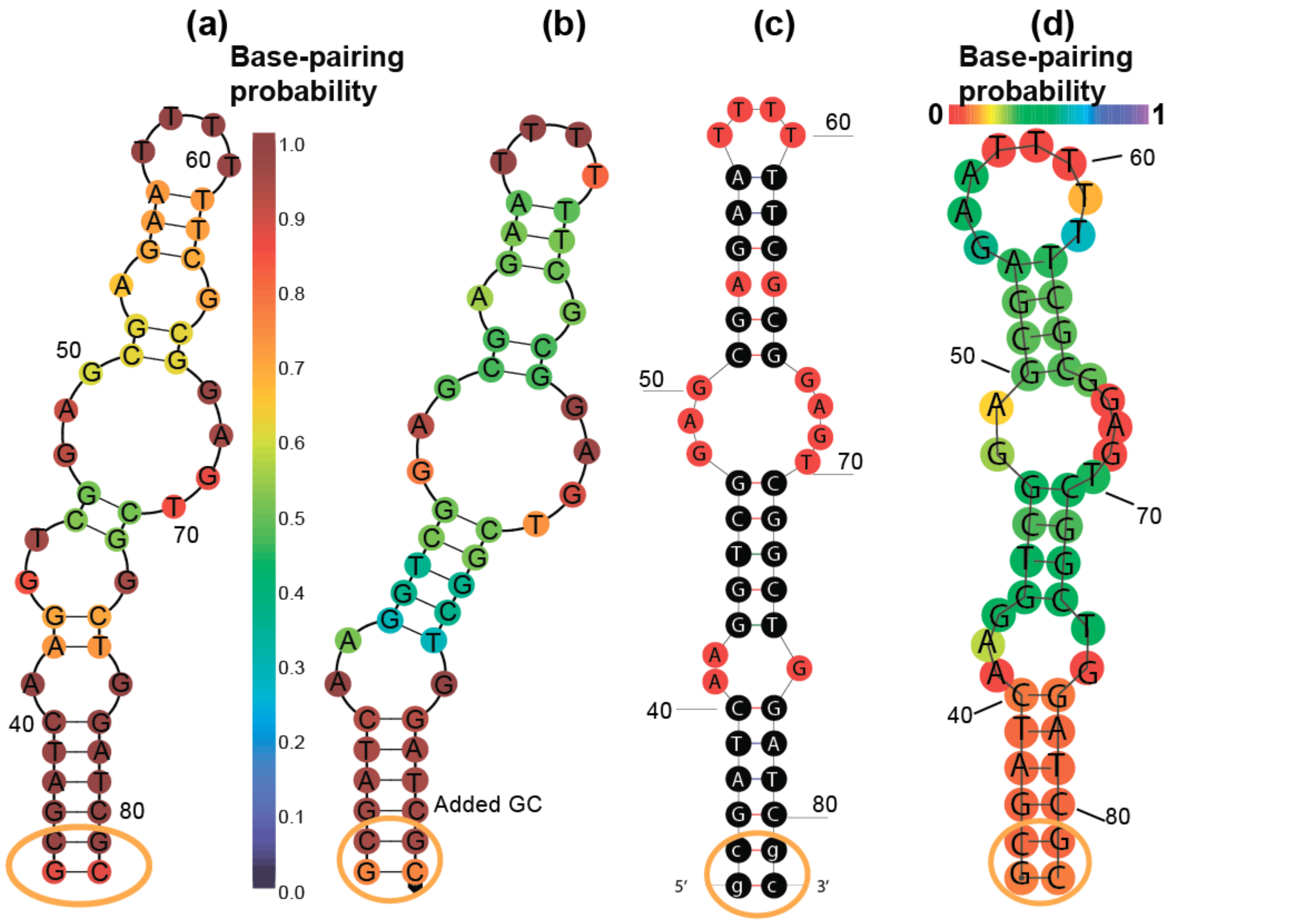

Figure S2. The 2D structures of aptamer s12_13_82m2 at 25 °C and the base-pairing probability obtained using: (a) UnaFold (Na=0.05, Mg = 0.003 M), (b) RNAfold (Total salt = 0.06), (c) NUPACK 4.2 and (d) NUPACK 4.0 (Na=0.05, Mg = 0.003 M).

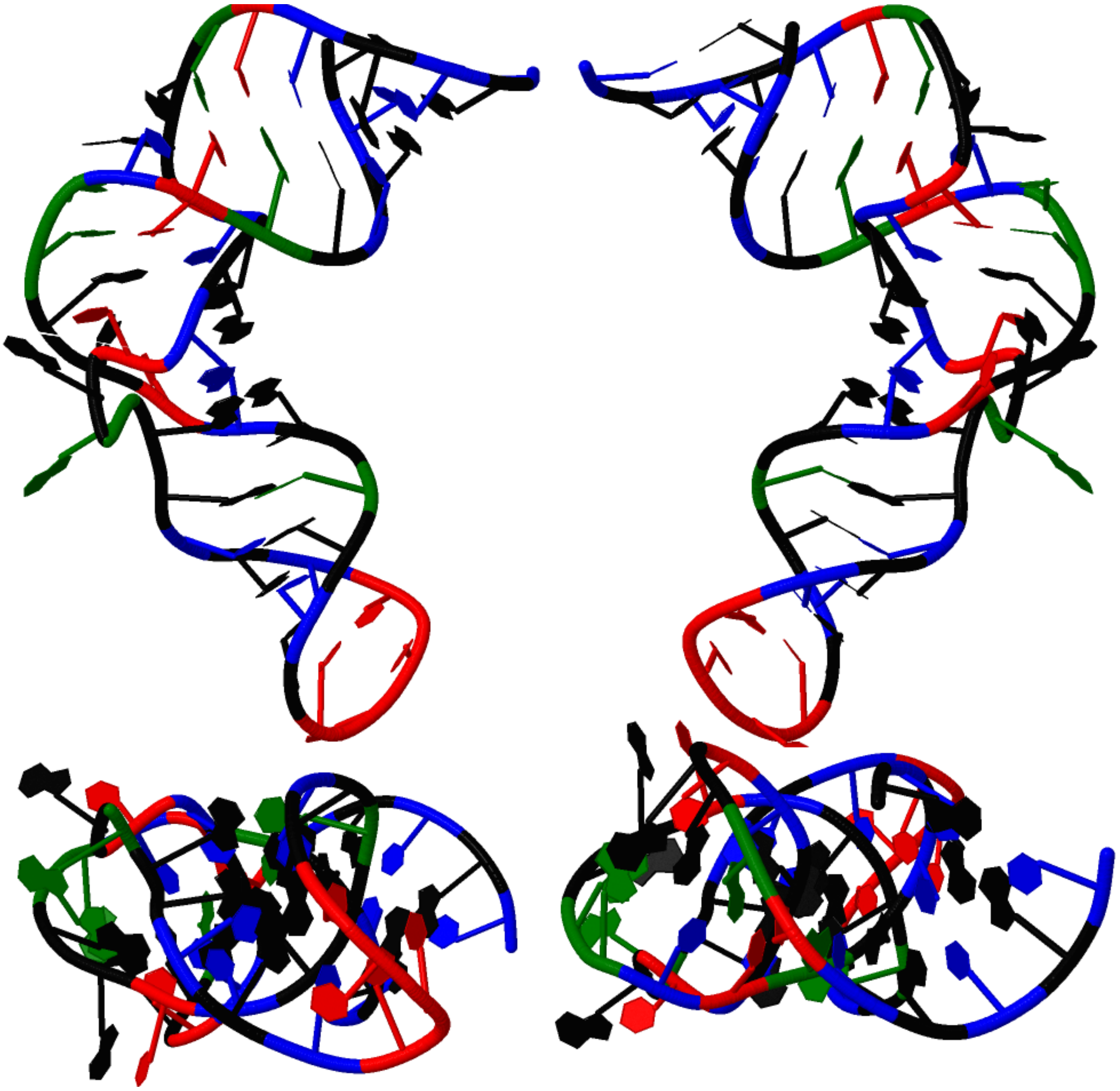

Figure S3. 3D structure of s12_13_82m7 predicted using RNAcomposer and visualized using w3DNA.

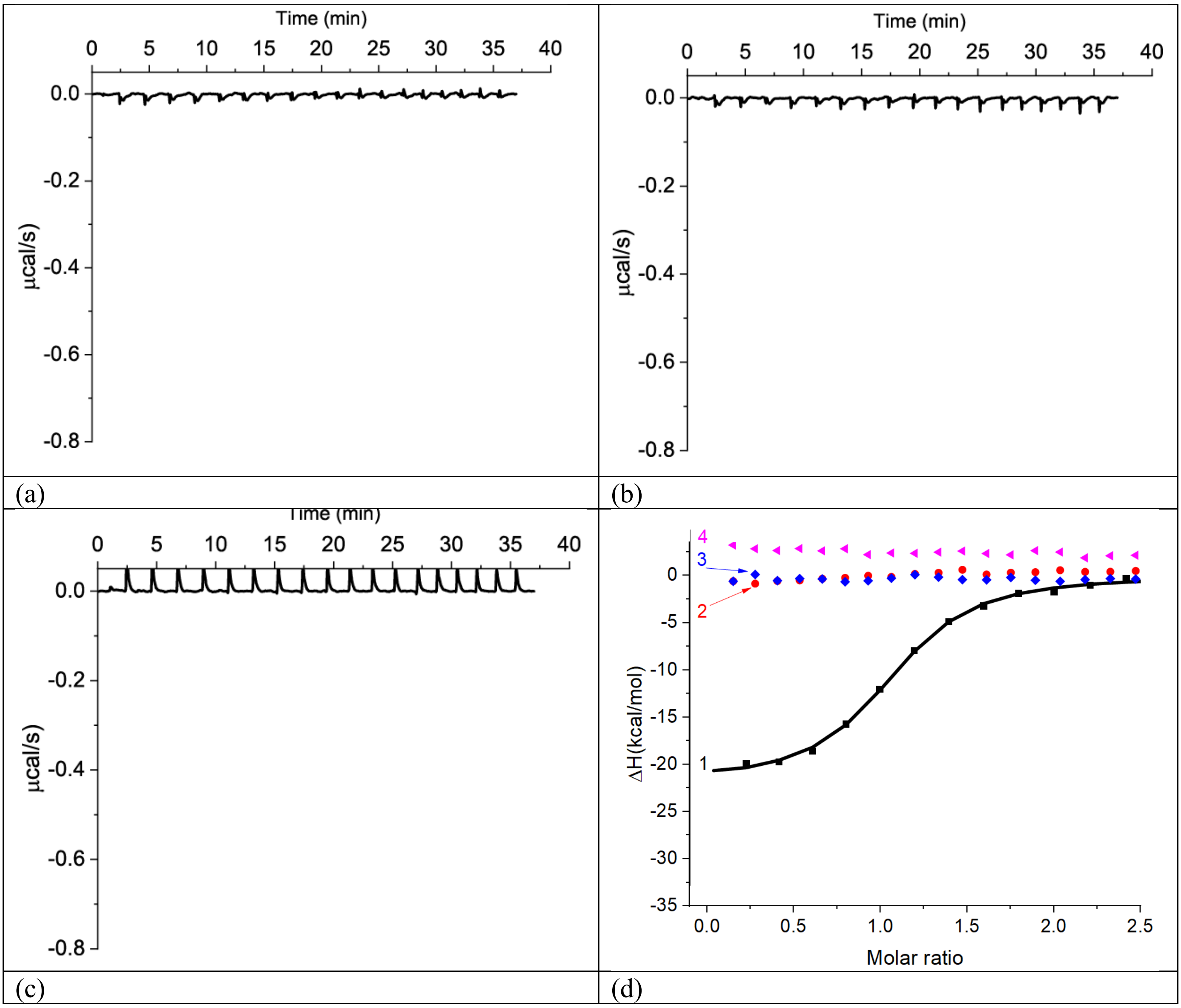

Figure S4. ITC measurements for sequential additions o150 μM of gentamicin (a,), neomycin(b), and streptomycin (c) to the 8 μM of s12_13_82 in BB. The comparison of integrated heat plots for interactions of s12_13_82 (d) with 1 – kanamycin (data from Fig.s1b), 2 – gentamicin, 3 – neomycin and 4 – streptomycin.

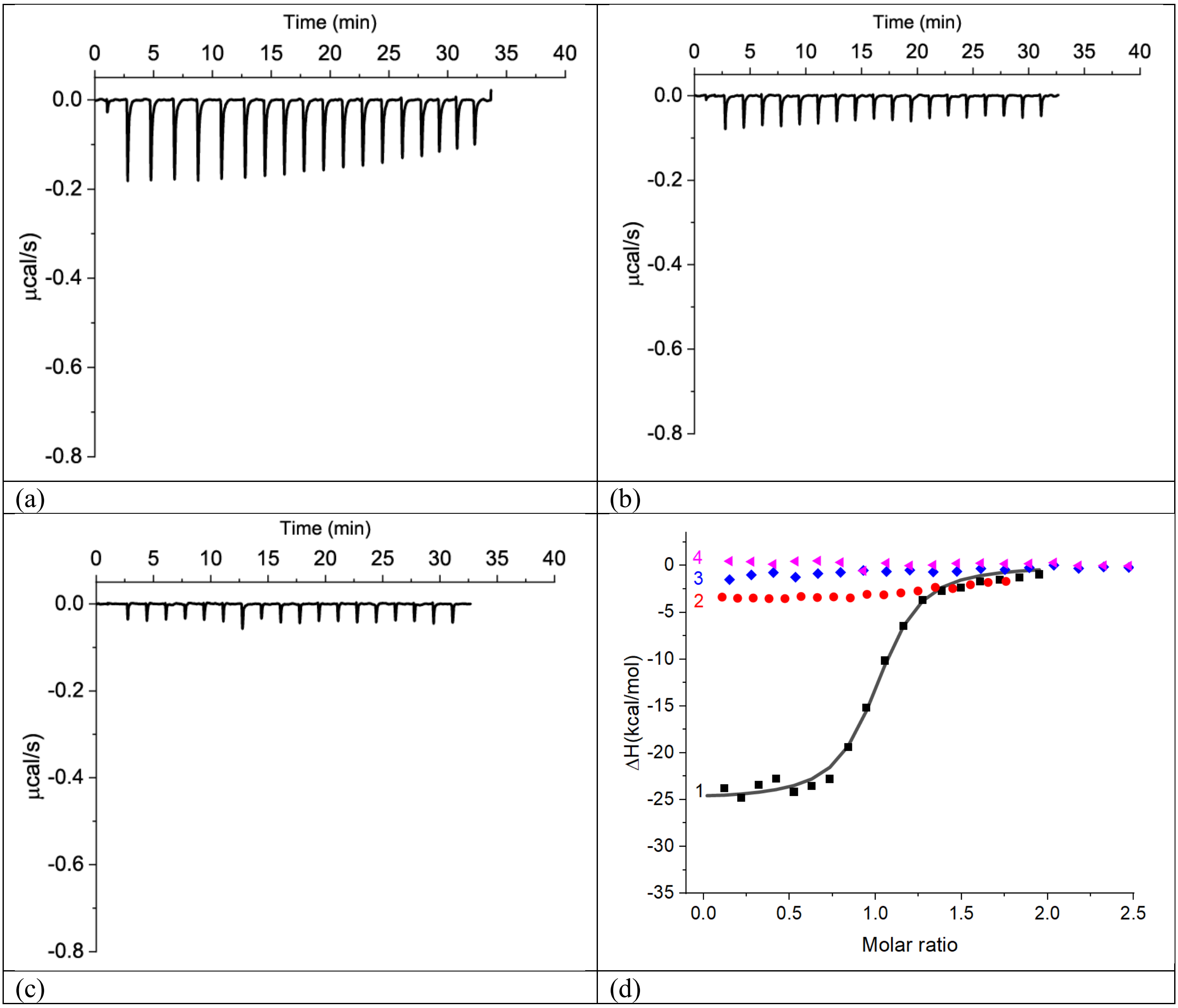

Figure S5 ITC measurements for sequential additions of 200µM of gentamicin (a), 150µM of neomycin(b), and streptomycin (c) to the 7.6 µM of s12_13_82m7 in BB. (d) The comparison of integrated heat plots for interactions of s12_13_82m7 with 1 – kanamycin (data from Fig.8c), 2 – gentamicin, 3 – neomycin and 4 – streptomycin.
